# Some Possible Clinical Units

**Published:** 1919-09-20

**Authors:** 


### SOME POSSIBLE CLINICAL UNITS.

It is not likely that the hospitals concerned—St. Bartholomew's, the "London," St. Thomas's, and University—will be able to start the Clinical Units in full swing by the October session, though perhaps a beginning will be made; in fact, Sir Cuthbert Wallace has been spoken of as the probable head of the Surgical Unit at St. Thomas's, and Mr. G. E. Gask of that at St. Bartholomew's Hospital. The Board of Education only made the definite offer at the end of July, and the holidays have been a bar to progress; there are also many points yet to be settled.

There is the question whether the head of the Unit should be required to give his whole time to the work, or only part time. In favour of whole time, it may be argued that medicine and surgery are such wide subjects that their teaching demands a man's whole time and energy, and that he should be free from the worry and distraction of private practice. Theoretically this may be sound, but practically, if whole time is demanded the field of candidates for the post will be limited—restricted, in fact, to young and untried men. A race of professors cannot be called into being in a moment. The most that could be paid to a professor would be about £2,500 a year, and few physicians or surgeons of eminence could give up all his private practice for this salary; present financial obligations would render it impossible without private means. Moreover, it is questionable whether it is fair to ask anyone to give up all private practice for the post, since it is very doubtful if the Board of Education can give an undertaking to finance the schemes for a period of even five years, especially when economy and retrenchment are so much to the fore; probably the grants will only be made from year to year. A professor at the end of five years might find himself deprived of his post and without any private practice or means of livelihood.

In favour of part time it may be argued that it is expedient that teachers of clinical work should keep in with private practice, and that a professor wholly engaged at a hospital is in danger of losing touch and sympathy with the practice of medicine or surgery in surroundings other than those of the hospital.

The hospitals will doubtless insist on the professors of the units taking their share with the other members of the staff in the ordinary routine work; otherwise the result might be the cutting off of a certain number of beds for educational purposes solely, and the number available for the treatment of the sick is none too many as it is.

It is not anticipated that the Board of Education will interfere in the actual appointment of the candidates, but the giving of the grant will be conditional on approval of the professor and his assistants.

Whether assistants will be whole or part time remains yet to be seen. The same objections to whole time apply as in the case of the professors. A first assistant's salary whole time might be £800 a year, and it is doubtful if this would attract the best men. The war has caused a gap of years in young men's careers, and a man of thirty-five with a good record is not likely to accept a post of £800, with the prospects as to where it will lead uncertain.

